# Patterns of Residential Segregation

**DOI:** 10.1371/journal.pone.0157476

**Published:** 2016-06-17

**Authors:** Rémi Louf, Marc Barthelemy

**Affiliations:** 1 Institut de Physique Théorique, CEA, IPhT, CNRS-URA 2306, F-91191 Gif-sur-Yvette, France; 2 Centre for Advanced Spatial Analysis, University College London, London, W1N 6TR, United Kingdom; 3 Centre d’Analyse et de Mathématique Sociales, EHESS, 190-198, avenue de France, 75244 Paris Cedex 13, France; Peking University, CHINA

## Abstract

The spatial distribution of income shapes the structure and organisation of cities and its understanding has broad societal implications. Despite an abundant literature, many issues remain unclear. In particular, all definitions of segregation are implicitely tied to a single indicator, usually rely on an ambiguous definition of income classes, without any consensus on how to define neighbourhoods and to deal with the polycentric organization of large cities. In this paper, we address all these questions within a unique conceptual framework. We avoid the challenge of providing a direct definition of segregation and instead start from a definition of what segregation is not. This naturally leads to the measure of representation that is able to identify locations where categories are over- or underrepresented. From there, we provide a new measure of exposure that discriminates between situations where categories co-locate or repel one another. We then use this feature to provide an unambiguous, parameter-free method to find meaningful breaks in the income distribution, thus defining classes. Applied to the 2014 American Community Survey, we find 3 emerging classes—low, middle and higher income—out of the original 16 income categories. The higher-income households are proportionally more present in larger cities, while lower-income households are not, invalidating the idea of an increased social polarisation. Finally, using the density—and not the distance to a center which is meaningless in polycentric cities—we find that the richer class is overrepresented in high density zones, especially for larger cities. This suggests that density is a relevant factor for understanding the income structure of cities and might explain some of the differences observed between US and European cities.

## Introduction

Challenges posed by the constantly growing urbanisation are complex and difficult to handle. They range from the increasing dependence on energy, to serious environmental and sustainability issues, and socio-spatial inequalities [1]. In particular, we observe the appearance of socially homogeneous zones and dynamical phenomena such as urban decay [2, 3] and gentrification [4] that reinforce the heterogeneity of the spatial distribution of social classes in cities. Such a segregation – characterized by an important social differentiation of the urban space – has significant social, economic [5] and even health costs [6] which justify the attention it has attracted in academic studies over the past century. Despite the abundant literature in sociology and economics, however, there is no consensus on the adequate way to quantify and describe patterns of segregation. In particular, the identification of neighbourhoods where the different groups gather is still in its infancy.

As stated many times, and at different periods in the sociology literature [7–10], the study of segregation is cursed by its intuitive appeal. The perceived familiarity with the concept favours what Duncan and Duncan [7] called ‘naive operationalism’: the tendency to force a sociological interpretation on measures that are at odds with the conceptual understanding of segregation. As a matter of fact, segregation is a complex notion, and the literature distinguishes several conceptually different dimensions. Massey [9] first proposed a list of 5 dimensions (and related existing measures), which was recently reduced to 4 by Reardon [11]. (i) *exposure* which measures the extent to which different populations share the same residential areas; (ii) the *evenness* (and *clustering*) to which extent populations are evenly spread in the metropolitan area; (iii) *concentration* to which extent populations concentrate in the areal units they occupy; and (iv) *centralization* to which extent populations concentrate in the center of the city.

We identify several problems with this picture. The first—fundamental – issue lies in the lack of a general conceptual framework in which all existing measures can be interpreted. Instead, we have a patchwork of seemingly unrelated measures that are labelled with either of the aforementioned dimensions. Although segregation can indeed manifest itself in different ways, it is relatively straightforward to define what is *not* segregation: a spatial distribution of different categories that is undistinguishable from a uniform random situation (with the same percentages of different categories). Therefore, we can define segregation as *any pattern in the spatial distribution of categories that deviates significantly from a random distribution* [12]. The different dimensions of [9, 11] then correspond to particular aspects of how a multi-dimensional pattern can deviate from its randomized counterpart. The measures we propose here are all rooted in this general definition of segregation.

The other issues are technical in nature. First, several difficulties are tied to the existence of many categories in the underlying data. Historically, measurements of racial segregation were limited to measures between 2 population groups. However, most measures generalise poorly to a situation with many groups, and the others do not necessarily have a clear interpretation [10]. Worse, in the case of groups based on a continuum (such as income), the thresholds chosen to define classes are usually arbitrary [13]. We propose in the following to solve this issue by defining classes in a unambiguous and non-arbitrary way through their pattern of spatial interaction. Applied to the distribution of income categories in US cities, we find 3 emergent categories, which are naturally interpreted as the lower-, middle- and higher-income classes. Second, most authors systematically design a single index of segregation for territories that can be very large, up to thousands of square kilometers [14]. In order to mitigate segregation, a more local, spatial information is however needed: local authorities need to locate where the poorest and richest concentrate if they want to design efficient policies to curb, or compensate for the existing segregation. In other words, we need to provide a clear *spatial* information on the pattern of segregation. Previous studies [15–18] were interested in the characterisation of intra-urban segregation patterns, but they suffer from the limitations of the indicators they use. In particular, the values they map come with no indication as to when a high value of the index indicates high segregation levels. As a result, the maps are not necessarily easy to read. Furthermore, all the descriptions are cartographic in nature and while maps are a powerful way to highlight patterns, we would like to provide further, quantitative, information about the spatial distribution that goes beyond cartographic representation.

The lack of a clear characterization of the spatial distribution of individuals is not tied to the problem of segregation in particular, but pertains to the field of spatial statistics [19–21]. Many studies avoided this spatial problem by assuming implicitely that cities are monocentric and circular, and rely on either an arbitrary definition of the city center boundaries, or on indices computed as a function of the distance to the center (whatever this may be). However, most if not all cities are anisotropic, and the large ones, polycentric (see [22] and references therein). Many empirical studies and models in economics aim to explain the difference between central cities and suburbs [23, 24]. Yet, the sole stylized fact upon which they rely—city centers tend to be poorer than suburbs (in the US)—lacks a solid empirical basis.

In the first part of the paper, we define a null model—the unsegregated city—and define the representation, a measure that identifies significant local departures from this null case. We further introduce a measure of exposure that allows us to quantify the extent to which the different categories attract or repel one another. This exposure is the starting point for the non-parametric identification of the different social classes. In the second part, we define neighbourhoods by clustering adjacent areal units where classes are overrepresented and show that there an increased spatial isolation of classes as population size of cities grows. We also show that larger cities are richer in the sense that the wealthiest households tend to be overrepresented and the low-income underrepresented in large cities. Finally, we discuss how density is connected to the spatial distribution of income, and how to go beyond the traditional picture of a poor center and rich suburbs.

We focus here on the income distribution, using the data for the 2014 Core-Based Statistical areas. However, the methods presented in this paper are very general, and can be applied [25] to different geographical levels, to an arbitrary number of population categories, and to different variables such as ethnicity, education level, etc.

## 1 The importance of a null model

Most studies exploring the question of spatial segregation define measures before comparing their value for different cities. Knowing that two quantities are different is however not enough: we also have to know whether this difference is significant. In order to assess the significance of a result, we have to compare it to what is obtained for a reasonable null model.

### 1.1 Definitions

We assume that we have *T* areal units dividing the city and that individuals can belong to different categories. The elementary quantity is *n*_*α*_(*t*) which represents the number of individuals of category *α* in the unit *t*. The total number of individuals belonging to a category *α* is *N*_*α*_ and the total number of individuals in the city is given by *N* = ∑_*α*_ *N*_*α*_.

In the context of residential segregation, a natural null model is the *unsegregated city*, where all households are distributed at random in the city with the constraints that

- The total number *n*(*t*) of households living in the areal unit *t* is fixed (from data).
- The numbers *N*_*α*_ are given by the data;

The problem of finding the numbers {*n*_*α*_(1), …, *n*_*α*_(*T*)} in this unsegregated city is reminiscent of the traditional occupancy problem in combinatorics [26]. If we assume that for all categories *α*, we have *n*_*α*_(*t*) ≪ *n*(*t*), they are then distributed according to the multinomial denoted by *f*(*n*_*α*_(1), …, *n*_*α*_(*T*)), and the number of people of category *α* in the areal unit *t* is distributed according to a binomial distribution. Therefore, in an unsegregated city, we have

$$\begin{array}{c} E\left[n_{\alpha }\left(t\right)\right]=N_{\alpha }\;\frac{n(t)}{N} \end{array}$$ (1)

$$\begin{array}{c} \text{Var}\left[n_{\alpha }\left(t\right)\right]=N_{\alpha }\;\frac{n(t)}{N}(1-\frac{n(t)}{N}) \end{array}$$ (2)

The fundamental quantity we will use in the following is the *representation* of a category *α* in the areal unit *t*, defined as

$$\begin{array}{c} r_{\alpha }\left(t\right)=\frac{n_{\alpha }\left(t\right)/n\left(t\right)}{N_{\alpha }/N}=\frac{n_{\alpha }\left(t\right)/N_{\alpha }}{n(t)/N} \end{array}$$ (3)

The representation thus compares the relative population *α* in the areal unit *t* to the value that is expected in an unsegregated city where individuals choose their location at random. Or, equivalently, the representation compares the proportion of individuals *α* in the unit *t* to their proportion in the city as a whole.

In metropolitan areas, *N*_*α*_ is large compared to 1, and the distribution of the *n*_*α*_(*t*) can be approximated by a Gaussian with the same mean and variance. Therefore we have in the unsegregated case

$$\begin{array}{ccc} E[r_{\alpha }\left(t\right)] & = & 1\\ \text{Var}[r_{\alpha }\left(t\right)] & = & \sigma_{\alpha }{\left(t\right)}^{2}=\frac{1}{N_{\alpha }}[\frac{N}{n(t)}-1] \end{array}$$ (4)

An important merit of the representation is the possibility to define rigorously the notion of *over*-representation and *under*-representation of a category *α* in a geographical area. A category *α* is overrepresented (with a 99% confidence) in the geographical area *t* if *r*_*α*_(*t*) > 1 + 2.57*σ*_*α*_(*t*). A category *α* is underrepresented (with a 99% confidence) in the geographical area *t* if *r*_*α*_(*t*) < 1 − 2.57*σ*_*α*_(*t*). If the value *r*_*α*_(*t*) falls in between the two previous limits, the representation of the category *α* is not statistically different (at this confidence level) from what would be obtained if individuals were distributed at random. Existing measures output levels of segregation (typically a number between 0 and 1) but do not indicate whether these levels are *abnormally* high. To this respect, the representation is a significant improvement over previous measures.

Note that the above null model is reminiscent of the ‘counterfactuals’ used in the empirical literature on agglomeration economies [27–29]. Also, the expression of the representation Eq (3) is very similar to the formula used in economics to compute comparative advantages [30], or to the localisation quotient used in various contexts [14, 31]. To our knowledge, however, this formula has never been justified by a null model in the context of residential location. The representation allows to assess the significance of the deviation of population distributions from the unsegregated city. As we will show below, it is also the building block for measuring the level of repulsion or attraction between categories allowing us to uncover the different classes and to identify the neighbourhoods where the different categories concentrate. Last, but not least, the representation defined here does not depend on the category structure at the city scale, but only on the spatial repartition of individuals belonging to each category. This is essential in order to be able to compare different cities where the group compositions—or inequality – might differ. Inequality and segregation are indeed two separate concepts, and the way they are measured should be distinct from one another.

Finally, we would like to mention that using the uniform distribution as a null model can have implications broader than the study of residential segregation. Indeed, from a very abstract perspective, the study of residential segregation is the study of labelled objects in space. The methods presented here can therefore be applied to the study of the distribution of any object in space. In particular, it can be used to identify the locations in a territory where populations with different characteristics (not necessarily socio-economic) concentrate.

### 1.2 Attraction and repulsion of categories

Another shortcoming of the literature about segregation is the lack of indicator to quantify to what extent different populations attract or repel one another. Such a measure of attraction or repulsion is however important to understand the dynamics and scale (intensity of attraction/repulsion) of residential segregation.

Our indicator is inspired by the M-value first introduced by Marcon and Puech in the economics literature to measure the concentration of industries [28] and used as a measure of interaction between retail store categories in [32]. These authors were interested in measuring the geographic concentration of different type of industries. While previous measures (such as Ripley’s K-value) allow to identify departures from a random (Poisson) distribution, the M-value’s interest resides in the possibility to evaluate different industries’ tendency to co-locate. The idea, in the context of segregation is simple: we consider two categories *α* and *β* and we would like to measure to which extent they are co-located in the same areal unit. To quantify the tendency of households to co-locate, we measure the representation of the category *β* as witnessed on average by individuals in category *α*, and obtain the following quantity *E*_*αβ*_

$$\begin{array}{c} E_{\alpha \beta }=\frac{1}{N_{\alpha }}\sum_{t=1}^{T}n_{\alpha }\left(t\right)\;r_{\beta }\left(t\right) \end{array}$$ (5)

Although it is not obvious with this formulation, this measure is symmetric: *E*_*αβ*_ = *E*_*βα*_ (see S1 Text). Effectively, this ‘E-value’ in this context is a measure of exposure, according to the typology of segregation measures proposed in [9]. However, unlike the other measures of exposure found in the literature [33], we are able to distinguish between situations where categories attract (*E* > 1) or repel (*E* < 1) one another. In the case of an unsegregated city, every household in *α* sees on average *r*_*β*_ = 1 and we have *E*_*αβ*_ = 1. If populations *α* and *β* attract each other, that is if they tend to be overrepresented in the same areal units, every household *α* sees *r*_*β*_ > 1 and we have *E*_*αβ*_ > 1 at the city scale. On the other hand, if they repel each other, every household *α* sees *r*_*β*_ < 1 and we have *E*_*αβ*_ < 1 at the city scale. The minimum of the exposure for two classes *α* and *β* is obtained when these two categories are never present together in the same areal unit and then

$$\begin{array}{c} E_{\alpha \;\beta }^{min}=0 \end{array}$$ (6)

The maximum is obtained when the two classes are alone in the system (see S1 Text for more details) and in this case we get

$$\begin{array}{c} E_{\alpha \;\beta }^{max}=\frac{N^{2}}{4N_{\alpha }N_{\beta }} \end{array}$$ (7)

In the case *α* = *β*, the previous measure represents the ‘isolation’ defined as

$$\begin{array}{c} I_{\alpha }=\frac{1}{N_{\alpha }}\sum_{t=1}^{t}n_{\alpha }\left(t\right)\;r_{\alpha }\left(t\right) \end{array}$$ (8)

and measures to which extent individuals from the same category interact which each other. In the unsegregated city, where individuals are indifferent to others when chosing their residence, we have $I_{\alpha }^{min}=1$. In contrast, in the extreme situation where individuals belonging to the class *α* live isolated from the others, the isolation reaches its maximum value

$$\begin{array}{c} I_{\alpha }^{max}=\frac{N}{N_{\alpha }} \end{array}$$ (9)

Of course, in order to discuss the significance of the values of exposure and isolation, one needs to compute the variance of the exposure in the unsegregated situation defined earlier. The calculations for the variance as well as for the extrema are presented in S1 Text.

Finally, we note that co-location is not necessarily synonymous with interaction, as pointed out by Chamboredon [34], and we should rigorously talk about *potential* interactions. Nevertheless, in the absence of large scale data about direct interactions between individuals, co-location is the best proxy available.

## 2 Emergent social classes

### 2.1 Defining classes

Studies that focus on the definition of a single segregation index for cities as a whole can avoid the problem of defining classes, either by measuring the between-neighbourhood variation of the average income (examples are the standard deviation of incomes [13], the variance of logged incomes [35] and Jargowsky’s Neighbourhood Sorting Index [13]), or by integrating over the entire income distribution (for instance the rank-order information theory index defined in [36]). However, when they investigate the behaviour of households with different income and their spatial distribution, studies of segregation must be rooted in a particular definition of categories (or classes). Unfortunately, there is no consensus in the literature about how to separate households in different classes according to their income, and studies generally rely on more or less arbitrary divisions [37–39].

While in some particular cases grouping the original categories in pre-defined classes is justified, most authors do so for mere convenience reasons. However, as some sociologists have already pointed out [40], imposing the existence of absolute, artificial entities is necessarily going to bias our reading of the data. Furthermore, in the absence of recognized standards, different authors will likely have different definitions of classes, making the comparisons between different results in the literature difficult.

From a theoretical point of view, entities such as social classes do not have an existence of their own. Grouping the individuals into arbitrary classes when studying segregation is thus a logical fallacy: it amounts to imposing a class structure on the society before assessing the existence of this structure (which manifests itself by the differentiated spatial repartition of individuals with different income). Here, instead of imposing an arbitrary class structure, we let the class structure emerge from the data themselves. Our starting hypothesis is the following: *if there is such a thing as a social stratification based on income, it should be reflected in the households’ behaviours*: households belonging to the same class should tend to live together, while households belonging to different classes should tend to avoid one another. In other words, we aim to define classes using the way they manifest themselves through the spatial repartition of the different categories.

### 2.2 Finding breaks in the income distribution

We choose as a starting point the finest income subdivision given by the US Census Bureau (16 subdivisions) and compute the 16 × 16 matrix of *E*_*αβ*_ values for all cities. We then perform a hierarchical clustering on this matrix, succesively aggregating the subdivisions with the highest *E*_*αβ*_ values. The process, that we implemented in the Python library Marble [25], goes as follows:

1. Check whether there exists a pair *α*, *β* such that *E*_*αβ*_ > 1 + 10*σ* (i.e. two categories that attract one another with at least 99% confidence according to the Chebyshev inequality). If not, stop the agregation and return the classes;
2. If there is at least one couple satisfying (1), normalize all *E*_*αβ*_ values by their respective maximum values. Find then the pair *γ*, *β* whose normalized exposure is the maximum;
3. Aggregate the two categories *β* and *γ*;
4. Repeat the process until it stops.

In order to aggregate the categories at step 3, we need to compute the exposure between *δ* = *β* ∪ *γ* and any category *α*, as well as its variance. The corresponding calculations are presented in S1 Text.

We stress that the obtained classification does not rely on any arbitrary threshold. Indeed, we stop the aggregation process when the only classes left are indifferent (*E*_*αβ*_ = 1 with 99% confidence) or repel each other (*E*_*αβ*_ < 1 with 99% confidence).

### 2.3 The US income structure

**Fig 1:**
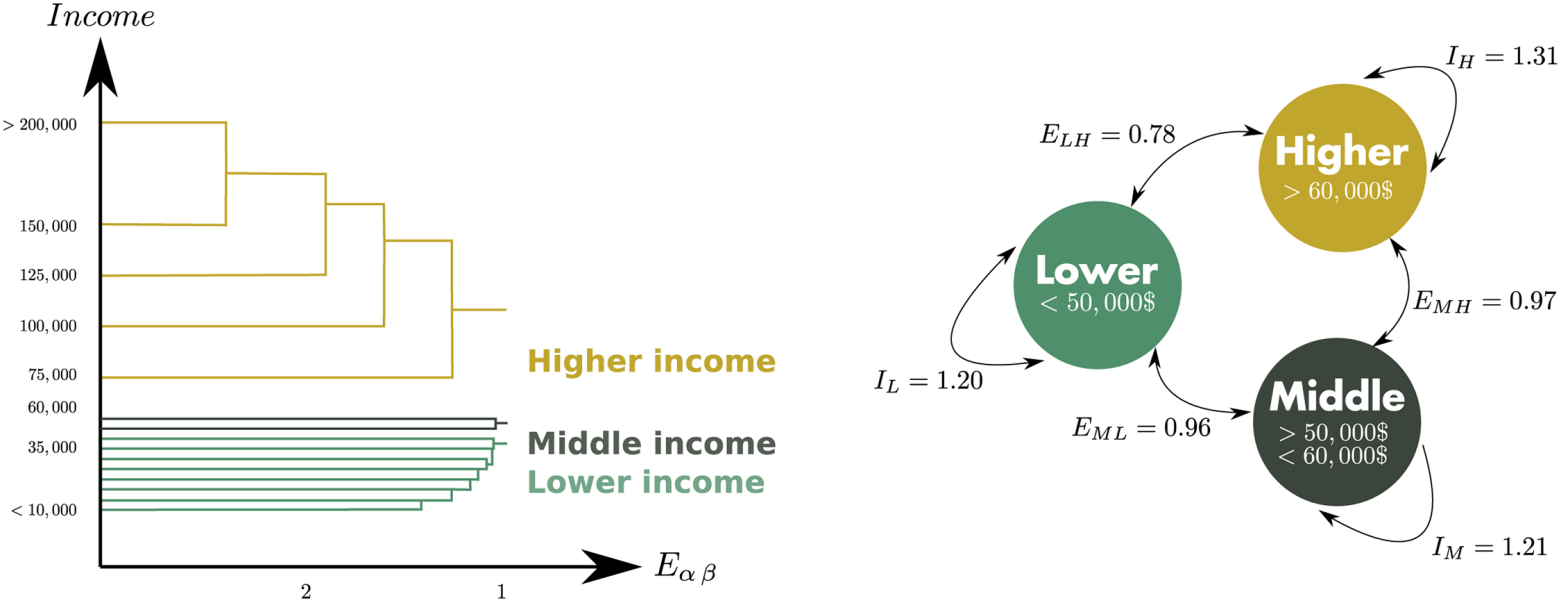
(Left) Alluvial diagram showing the successive aggregation of different income categories during the clustering process. The x-axis shows the value of the exposure at which each aggregation took place. The aggregation stops when there is no pair of category (*α*, *β*) for which *E*_*αβ*_ > 1, that is when all classes are at best indifferent to one another (see S1 Text, section 2 for a more detailed description of the algorithm). One can see on this diagram that the highest income categories tend to colocate more (higher values of *E*_*αβ*_) than the lowest income categories. (Right) The 3 classes that emerge from the clustering process. In the circles we indicate the range of income to which each class corresponds. The value next to the arrows correspond to the respective values of exposure and isolation. As the values of exposure show, the lower- and higher-income classes repel one another, while the middle-income class is indifferent to the other classes. Furthermore, the higher-income class is a more coherent group than the middle-income and lower-income classes, as reflected by the values of the isolation coefficient *I*.

Strikingly, the outcome of this method on US data is the emergence of 3 distinct classes (Fig 1): the higher-income (∼29% of the US population) and the lower-income classes (∼59% of the US population)—which repel each other strongly while being respectively very coherent—and a somewhat small middle-income class (∼11% of the population) that is relatively indifferent to the other classes. This result implies that there is some truth in the conventional way of dividing populations into 3 income classes, and that what we casually perceive as the social stratification in our cities actually emerges from the spatial interaction of people.

Our method has several advantages over a casual definition: it is not arbitrary in the sense that it does not depend on a tunable parameter (besides the significance threshold) and on who performs the analysis. Its origins are tractable, and can be argued on a quantitative basis. Because it is quantitative, it allows comparison of the stratification over different points in time, or between different countries. It can also be compared to other class divisions that would be obtained using a different medium for interaction, for instance mobile phone communications [41].

In the following, we will systematically use the classes obtained with this method.

### 2.4 Larger cities are richer

**Fig 2:**
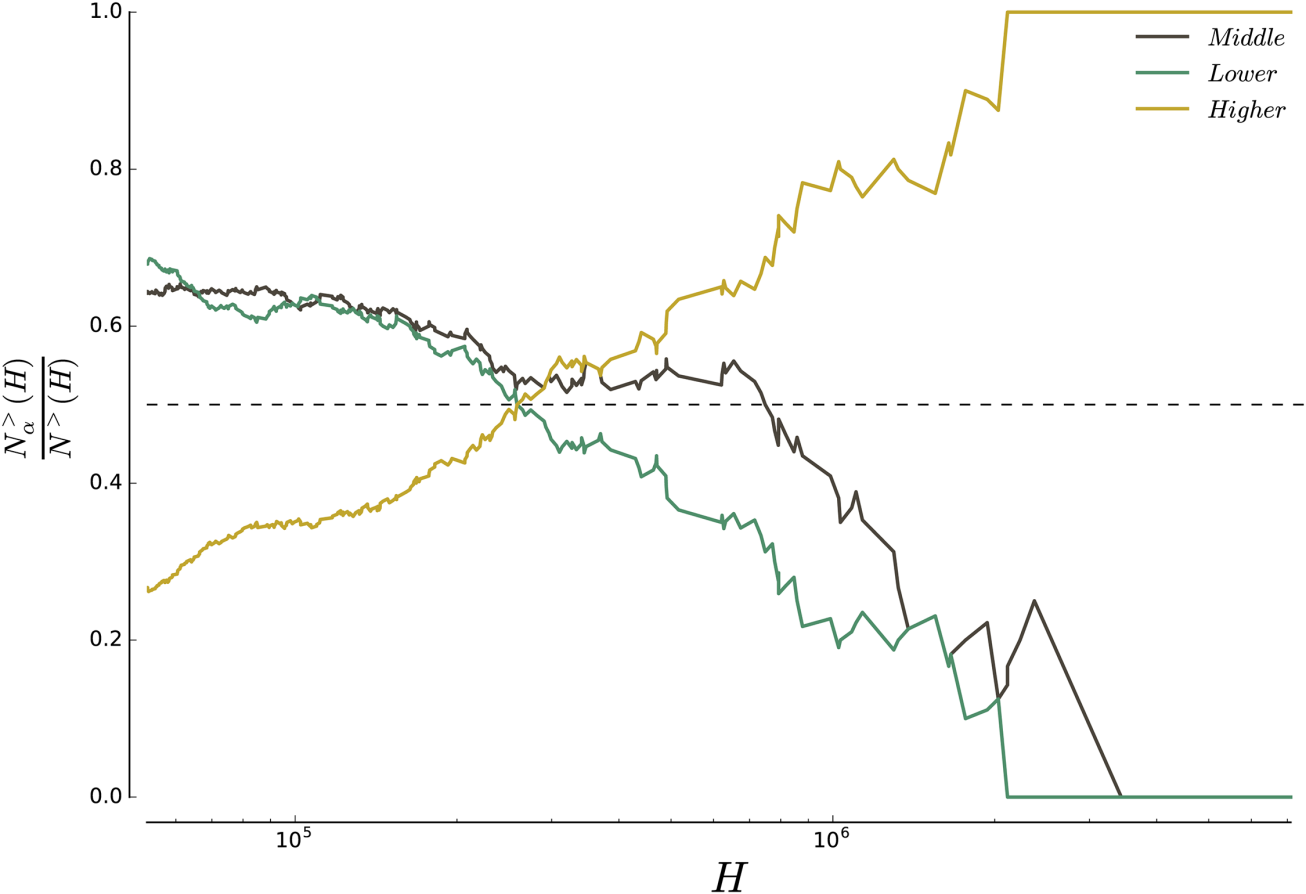
Proportion of cities in which the different classes are overrepresented, as a function of the total population of the city. One can clearly see that the larger the city, the more likely it is that the high-income class is overrepresented and the lower-income class underrepresented (compared to national levels). This proves that the different income classes are not homogeneously distributed across all cities in the country. There is also a clear influence of population size on the representation of the different classes at the city level.

At the scale of an entire country, segregation can manifest itself in the unequal representation of the different income classes across the urban areas. We plot on Fig 2 the ratio $N_{\alpha }^{>}\left(H\right)/N^{>}\left(H\right)$ where *N*^>^(*H*) is the number of cities of population greater than *H*, and $N_{\alpha }^{>}\left(H\right)$ the number of cities of population greater than *H* for which the class *α* is overrepresented. A decreasing curve indicates that the class *α* tends to be underrepresented in larger urban areas, while an increasing curve shows that the category *α* tends to be overrepresented in larger urban areas (the representation is here measured with respect to the total population at the US level). These results challenge Sassen’s thesis on social polarization, according to which world (very large) cities host proportionally more higher-income and lower-income individuals than smaller cities [42]. If this thesis were correct, we should observe an overrepresentation of both higher-income and lower-income households in larger cities. Instead, as shown on Fig 2, higher-income households are overrepresented in larger cities, while lower-income households tend to be underrepresented (see S1 Text for a detailed discussion). These results support the previous critique of the social polarisation thesis by Hamnett [43].

## 3 Characterizing spatial patterns

The representation measure introduced at the beginning of this article allows to draw maps of overrepresentation and thus to identify the areas of the city where categories are overrepresented. In the following, we propose to characterise the spatial arrangement of these areas for the different categories.

### 3.1 Poor center, rich suburbs?

#### 3.1.1 A density-based method

In many studies, the question of the spatial pattern of segregation is limited to the study of the center versus suburbs and is usually adressed in two different ways.

In the first case, a central area is defined by arbitrary boundaries and measures are performed at the scale of this central area and the rest is labelled as ‘suburbs’. The issue with this approach is that the conclusions depend on the chosen boundaries and there is no unique unambiguous definition of the city center: while some consider the Central Business District [23], others choose the urban core (urbanized area) where the population density is higher.

The second approach, in an attempt to get rid of arbitrary boundaries, consists in plotting indicators of wealth as a function of distance to the center [23, 44]. This approach, inspired by the monocentric and isotropic city of many economic studies such as the Von Thunen or the Alonso-Muth-Mills model [45], has however a serious flaw: cities are not isotropic and are spread unevenly in space, leading to very irregular shapes [46]. Representing any quantity versus the distance to a center thus amounts to average over very different areas and in polycentric cases (as it is the case for large cities [22]) is necessarily misleading. As we show below, this method mixes together areas that are otherwise very different.

We propose here a different approach that does not require to draw boundaries between the center and the suburbs. In fact, it does not even require to define and locate the ‘center’ at all. In the case of a monocentric and isotropic city, our method gives results similar to those given by the other measures. In the more general case where cities are not necessarily monocentric neither isotropic, our method allows to compare regions of equivalent densities.

**Fig 3:**
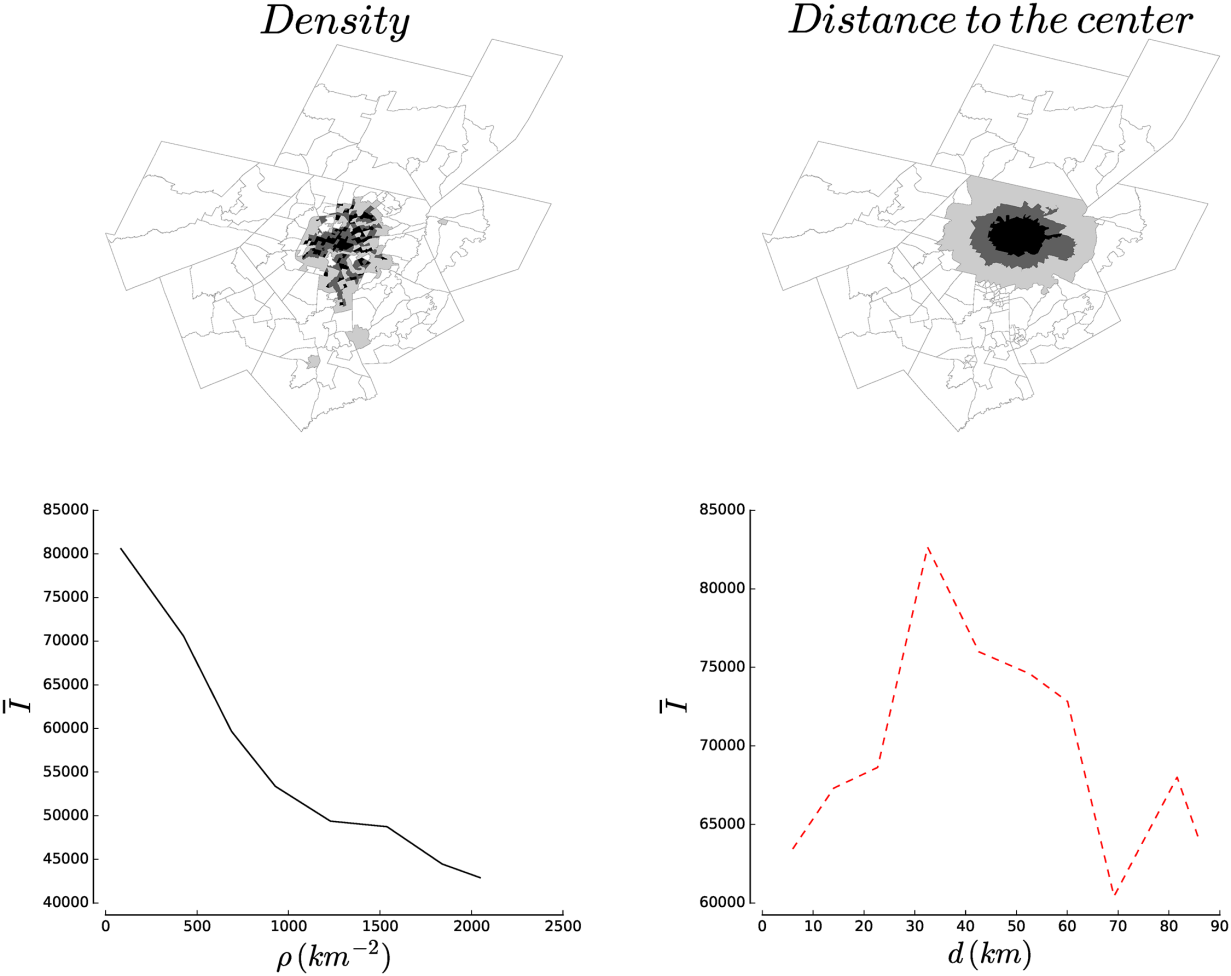
Illustrating the density approach. (Top Left) Blockgroups in Seattle, WA; the colour of a block depends on the population density interval to which it belongs (from the 25% most dense in black to the 25% least dense in white). (Top Right) Blockgroups in Seattle, WA coloured by distance to the center defined as densest blockgroup. The colour goes from black for the 25% closest blocks to white for the 25% closest ones. (Bottom Left) The average income of households as a function of density. (Bottom right) Average income of households as a function of distance to the center. Both methods give very different results.

The center of a city is usually defined as the region which has the highest population (or employment) density. We therefore propose the density as a proxy to measure of how ‘central’ an area is. We thus plot quantities computed over all areal units (blockgroups in this dataset) that have a density population in a given interval [*ρ*, *ρ* + *dρ*] where *ρ* decreases from its maximum to its minimum value. We illustrate this idea and compare its results to the traditional ‘distance to the center’ method on Fig 3. With very anisotropic and polycentric cities such as Los Angeles, the order in which the areal units are considered is very different with both methods. As a result, measurements as simple as the average income will yield very different results. This is particularly striking with the example of Seattle, WA shown on Fig 3. The average income as a function of the distance to the center (areal unit with the highest density) increases from the center to a peak at roughly 30 km (bottom left figure). On the other hand, the bottom left figure shows that average income is, in fact, a simple decreasing function of residential density.

Where does the discrepancy come from? As one can see on the maps on the top of Fig 3, the units considered in a given distance range can be very different in terms of density. Because real cities are neither monocentric or isotropic, units at a same distance from the center can in fact be very different. This shows, if anything, the importance of expliciting what one means by ‘central’ before presenting measures. In the following, we express centrality in terms of residential density.

#### 3.1.2 Results

**Fig 4:**
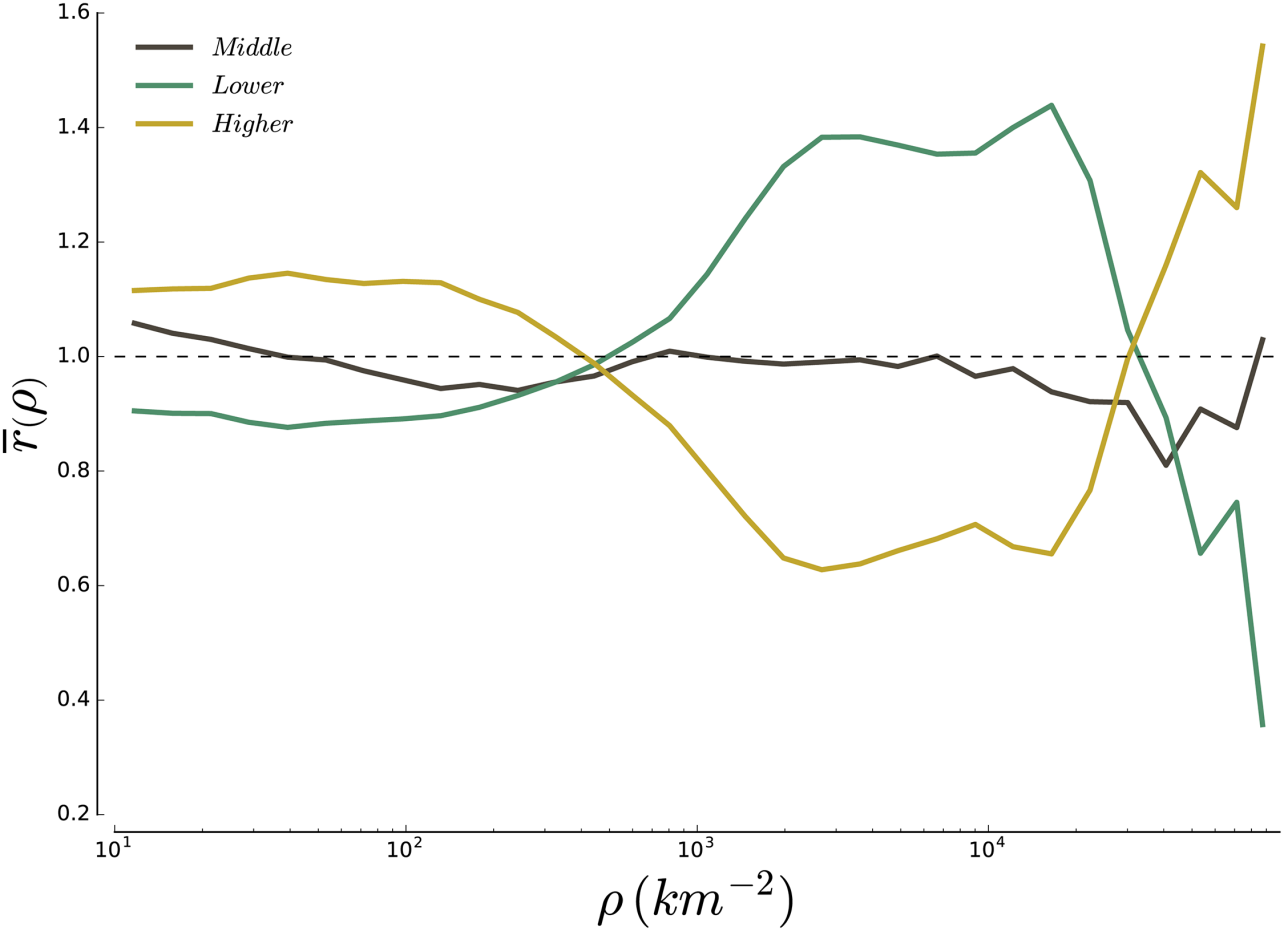
Average representation $\bar{r}$ of the higher-, middle- and lower-income classes over the 929 CBSA as a function of the local density of households. On average, we find that low-density regions (the suburbs) are on the richer end, while high density regions (the center) are on the poorer end. This confirms on a large dataset a stylized fact that had previously emerged from local studies. Interestingly, we also find that high income households are on average overrepresented in very large density areas (*ρ* > 20,000/*km*^2^), suggesting that density may be one relevant factor in the explanation of the differences between neighbourhoods.

Here, we compute the representation of groups as a function of residential density. This method sheds a new light on the difference of social composition between the high-density and low-density areas in cities. Indeed, as shown on Fig 4, we find that low-density regions in cities are on average rich neighbourhoods and that higher density regions are on average lower-income neighbourhoods, in agreement with the dichotomy rich suburbs/poor centers usually found in the literature. But the dichotomy is not the full picture. The method indeed entails a surprising result: areas with very large densities (typically above 10,000 inhabitant/km^2^) are on average rich neighbourhoods. Only few cities in the US have neighbourhoods that reach the threshold of 20,000 inhabitants per km^2^, which can explain why people have reported in most cases the existence of poor centers and rich suburbs. In fact, among all 630 blockgroups with a population density greater than 20,000 inhabitants, 91% are located in New York, NY. Most high-density blockgroups belonging to other metropolitan areas exhibit an overrepresentation of the higher-income group and it is thus difficult to conclude at this stage that we are observing an effect specific to Manhattan. In any case, this result suggests that density is very relevant in the usual discussion about income strucutre differences between north american and european cities.

### 3.2 Neighbourhoods and their properties

**Fig 5:**
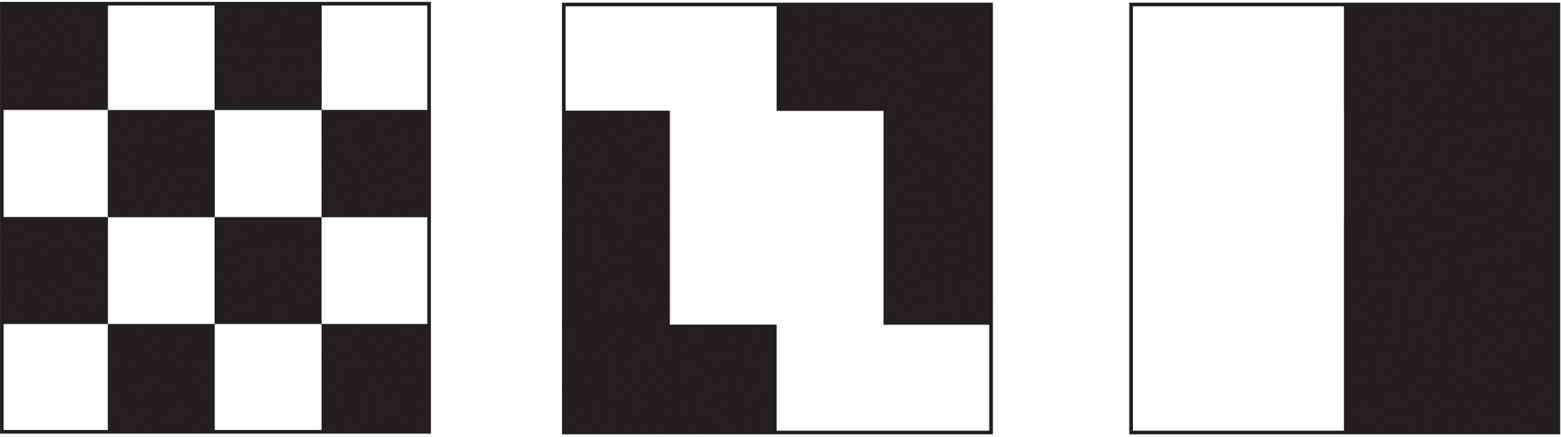
Three (hypothetical) spatial distributions of households that lead to the same values for intra-areal unit measures, but represent different segregation patterns. (Left) The checkerboard city popularised by White [47] corresponds to a clustering value—defined in Eq (10)—of *C* = 0 for the black squares. (Middle) An intermediate situation between the checkerboard and the divided city, corresponding to *C* ≈ 0.86. (Right) The divided city, corresponding to *C* = 1.

Intra-unit measures such as the representation or the exposure are not enough to quantify segregation. Indeed, areal units where a given class is overrepresented can arrange themselves in different ways, without affecting intra-unit measures of segregation [47]. In order to illustrate this, we consider the schematic cases represented on Fig 5, and assume that they are obtained by reshuffling the various squares around. Obviously, the checkerboard on the left depicts a very different segregation situation from the divided situation on the right while intra-unit measures would give identical results.

#### 3.2.1 Defining neighbourhoods

Defining neighbourhoods in which categories tend to gather is a difficult task. Indeed, what individuals call neighbourhoods often depend on their perception of the city, and field work is often necessary to identify which areas are socially recognised as being a large, middle or low income neighbourhood. However, it is often not possible to do field work and finding a way to define neighbourhoods from population counts is by more convenient and reliable.

**Fig 6:**
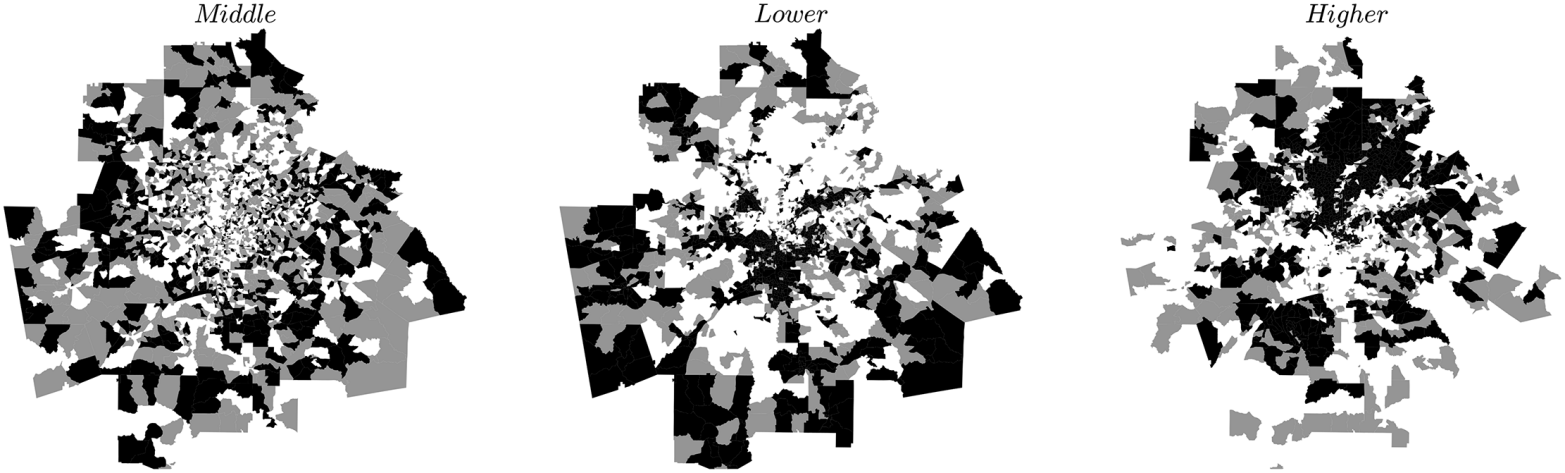
The neighbourhoods in Atlanta for the three income classes. In black, the blockgroups where the corresponding class is overrepresented; in white, where it is underrepresented; in grey, where the value of the representation is not distinguishable from the value that would be obtained if households chose their residence at random. It is interesting to note that all CBSA defined for the 2014 American Community Survey exhibit a total exclusion between lower-income and higher-income neighbourhoods: the pictures for lower- and higher-income classes are the perfect negative of one another. In contrast, middle-income households are scattered across the city and exhibit very little geographical coherence.

What is usually defined as a neighbourhood defies the most naive measures. For instance, to be a member of an ‘*α* neighbourhood’ (where *α* is here higher, middle or low income class) it is not necessary for an area to have a majority of individuals from the class *α* [48]. More sophisticated methods are thus required and the literature is not exempt of such measures, that are all rooted in different assumptions about the nature of neighbourhoods [48–51]. For instance, Logan et al. [48] use local K-functions in order to assess the prevalence of individuals from a class in an area. The areas are then clustered using a standard k-means clustering algorithm. The main issue with this approach is the use of K-functions which measure absolute concentration and are based on the null hypothesis of a completely random distribution of individuals across space. As mentioned earlier in this manuscript, it is more accurate to consider deviations from the null hypothesis of a random distribution of individuals *among existing locations*. We thus propose here an improvement over Logan et al.’s definition based on data given at the areal unit level (but could easily be generalised to data with exact locations). As in [48], we start with the intuitive idea that an *α* neighbourhood is an area of the city where the category *α* is more present than in the rest of the city. In other words, an areal unit *t* belongs to an *α* neighbourhood if and only if the category *α* is overrepresented in *t*, i.e. *r*_*α*_(*t*) > 1. We then build neighbourhoods by aggregating the adjacent areal units where the income class *α* is also overrepresented (see for example of Atlanta Fig 6).

#### 3.2.2 Clustering

**Fig 7:**
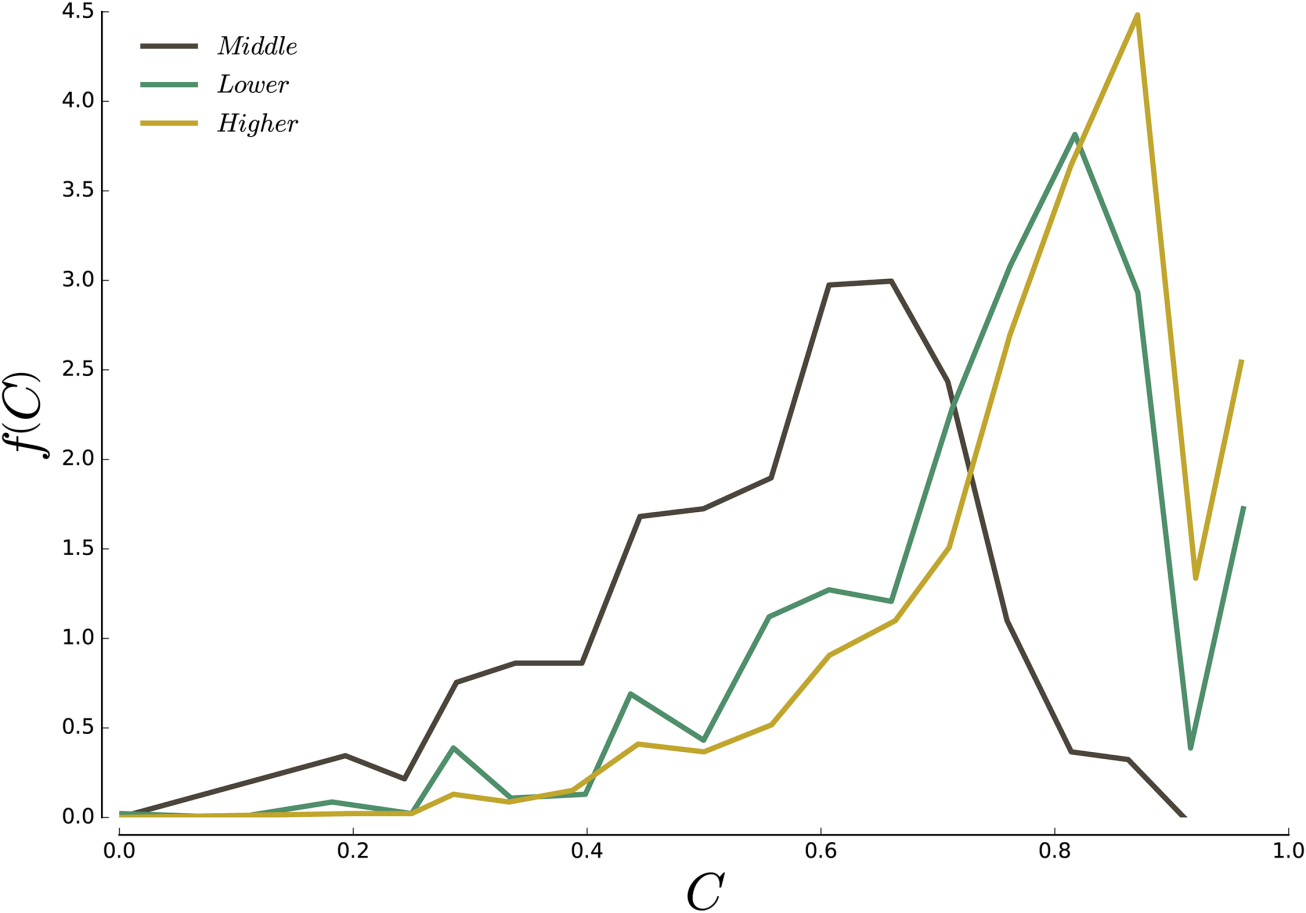
Distribution *f*(*C*) of the value of the clustering coefficient *C* per class for all cities in our dataset. The higher-income class exhibits the highest level of clustering, with an average of $\bar{C}=0.83$, followed by the lower-income class with on average $\bar{C}=0.78$. The middle-income class households are significantly less clustered than the other two, with $\bar{C}=0.52$ on average. The average is computed over all US core-based statistical areas.

A way to distinguish between different spatial arrangements is to measure how clustered the overrepresented areal units are. The ratio of the number *N*_*n*_(*α*) of *α*-neighbourhoods (clusters) to the total number *N*_*o*_(*α*) of areal units when the class *α* is overrepresented (before constructing the neighbourhood as defined above) gives a measure of the level of clustering and the quantity

$$\begin{array}{c} C_{\alpha }=\frac{N_{o}\left(\alpha \right)-N_{n}\left(\alpha \right)}{N_{o}\left(\alpha \right)-1} \end{array}$$ (10)

is such that *C*_*α*_ = 0 in a checkerboard-like situation, and *C*_*α*_ = 1 when all areal units form a unique neighbourhood. We show on Fig 7 the distribution of *C*_*α*_ for the three classes over all cities in our dataset. As one could infer from the maps on Fig 6, the higher-income and lower-income areal units are well clustered, with a respective average clustering of *C* = 0.83 and *C* = 0.78. The middle class is on the other hand less spatially coherent, with a average clustering *C* = 0.52.

#### 3.2.3 Concentration

**Fig 8:**
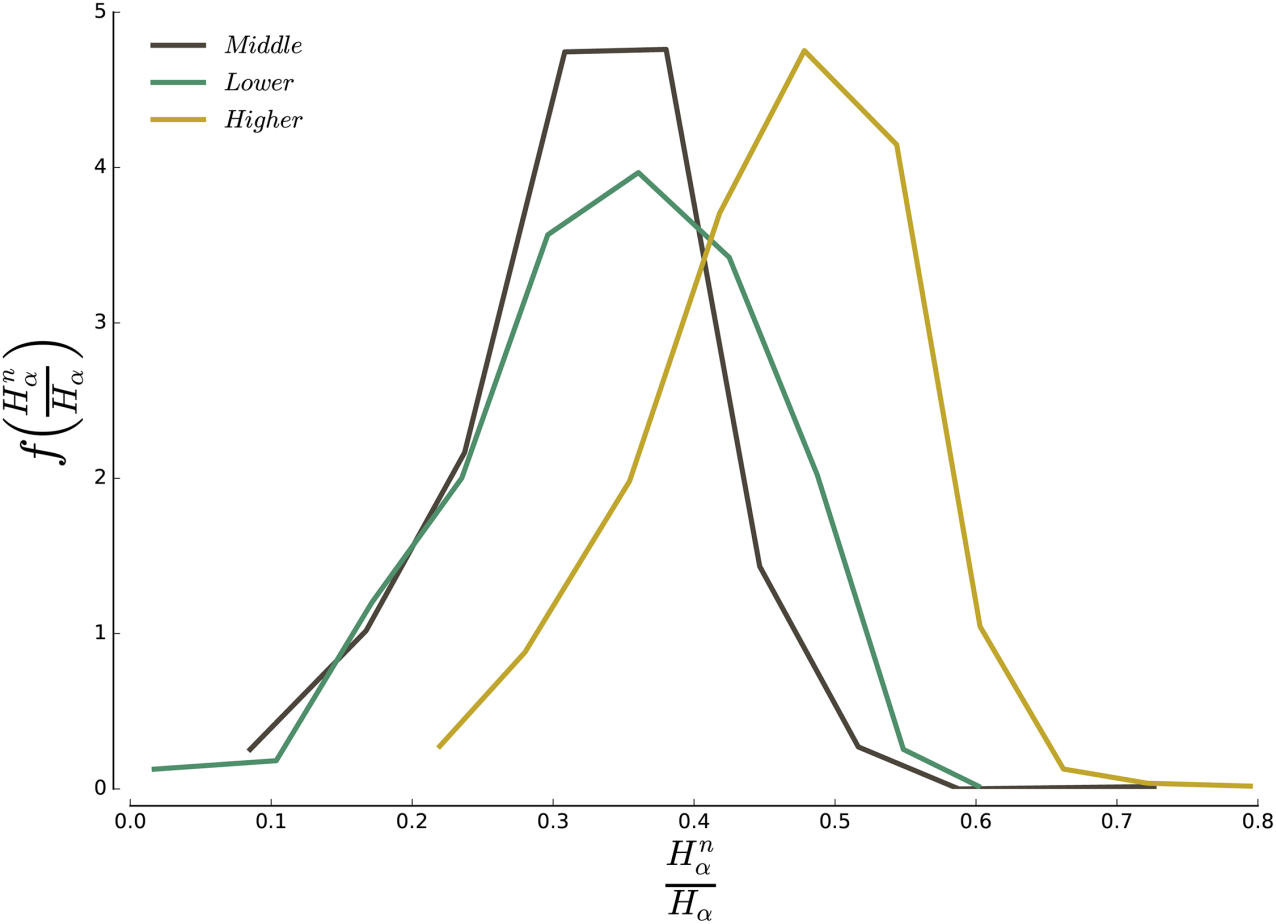
Concentration in neighbourhoods. For each class *α* we compute the fraction of households $H_{\alpha }^{n}/H_{\alpha }$ that belongs to an *α* neighbourhood. The figure shows the distribution of this fraction for all 2014 CBSAs.

If a given class is overrepresented in a neighbourhood, it does not necessarily mean that most of the individuals belonging to this neighbourhood are members of this class [48]. Conversely, the majority of individuals belonging to a class do not necessarily all live in the above-defined neighbourhoods. We thus compute the ratio of households of each income class that lives in a neighbourhood over the total number of individuals for that income class (rich, poor, and middle class). Our results (Fig 8) indicate that about 50% of the households belonging to *α* live in a *α*-neighbourhood, while the rest is dispatched across the rest of the city. The average concentration decreases from higher-income households (52%), to lower-income (40%) and middle-income household (40%).

#### 3.2.4 Fragmentation

**Fig 9:**
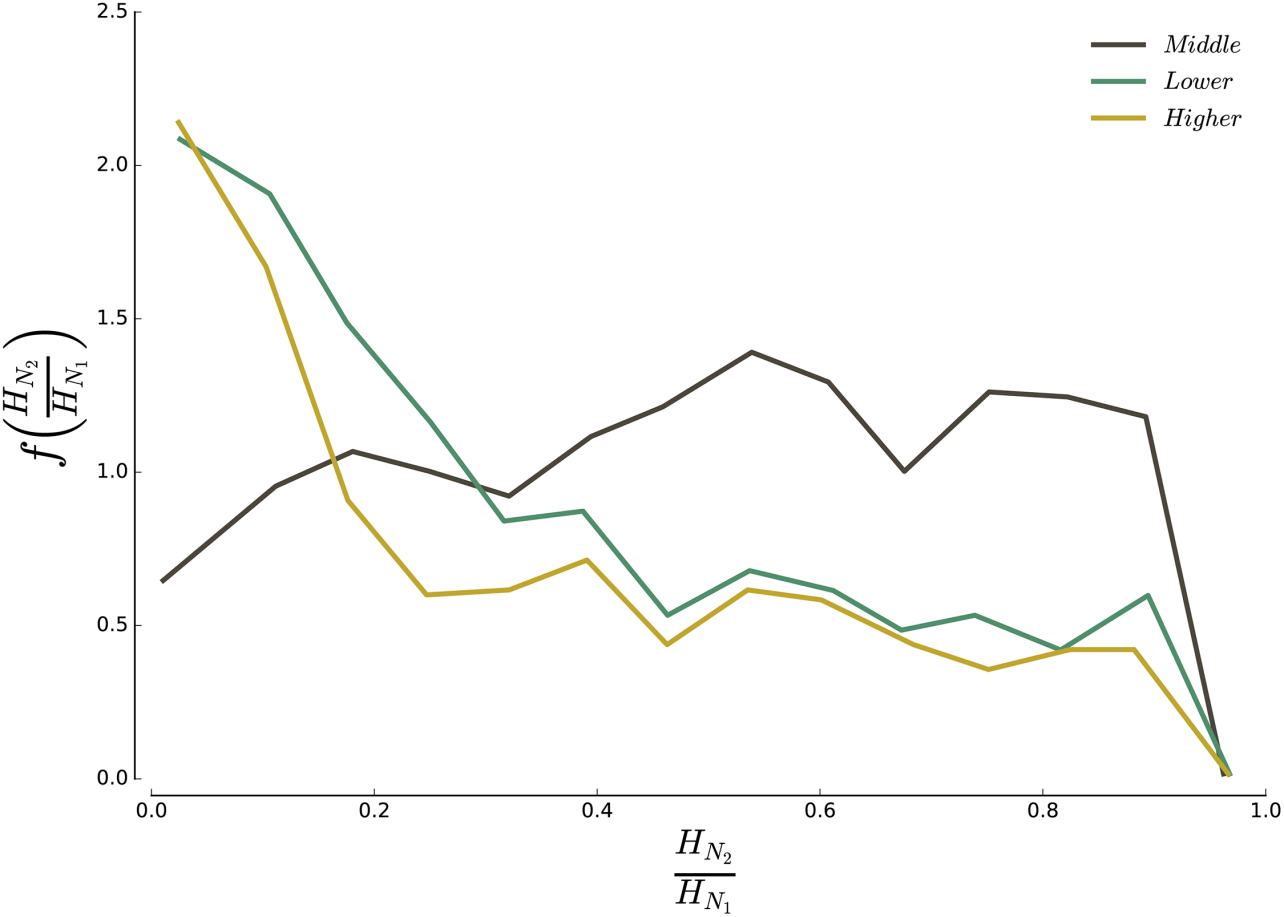
Neighbourhood fragmentation. For each class *α* we compute the ratio of the size of the second largest *α*-neighbourhood to the size of the largest *α*-neighbourhood. The above figure shows the distribution of this ratio for all cities in our dataset. Higher- and lower-income households tend to concentrate in a single neighbourhood, with a secondary center that is respectively 27% and 33% the size of the largest one, on average over all cities. Middle-income households tend to be more dispersed, with a secondary neighbourhood that is on average 54% of the size of the largest.

Finally, large values of clustering can hide different situations. We could have on one hand a ‘giant’ neighbourhood and several isolated areal units, which would essentially mean that each class concentrates in a unique neighbourhood. On the other hand, we could observe several neighbourhoods of similar sizes, meaning that the different classes concentrate in several neighbourhoods across the city. In order to distinguish between the two situations, we plot

$$\begin{array}{c} P=H_{N_{2}}/H_{N_{1}} \end{array}$$ (11)

where *H*_*N*_1__ is the population of the largest neighbourhood, and *H*_*N*_2__ the population of the second largest neighbourhood. The results are shown on Fig 9, and again display a different behaviour for the middle-income on one side, and higher-income and lower-income on the other side. The size of the middle-income neighbourhoods are more balanced, with on average *P* = 0.54. In contrast, higher- and lower-income neighbourhoods are dominated by a single large neighbourhood with on average *P* = 0.27 and *P* = 0.33, respectively.

### 3.3 Larger cities are more segregated

**Fig 10:**
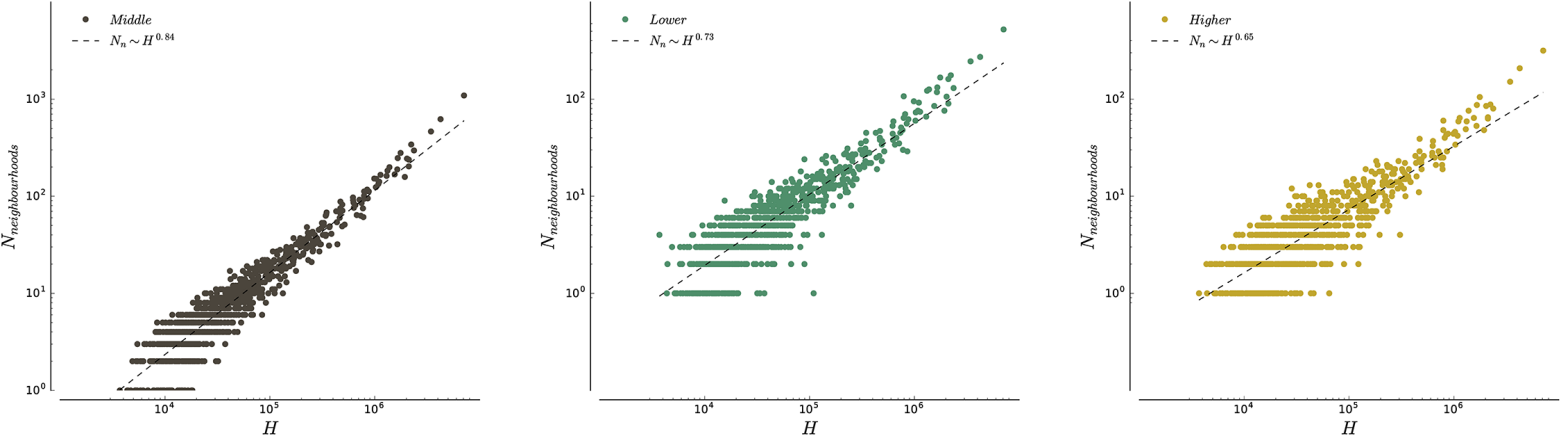
Number of neighbourhoods for the three different classes as a function of the size of the city. These plots in log-log show that we have a behavior consistent with a power law with exponent less than one (and with different value for each class). Combined with the linear increase of the number of over-represented units with the number of households (see S1 Text), this sublinear increase in the number of neighbourhoods shows the tendency of classes to cluster more as cities get larger.

As seen in Fig 7, the clustering values are high, indicating that the neighbourhoods occupied by households of different classes are sound. We can now wonder whether there is an effect of the city size on the number of neighbourhoods. We plot on Fig 10 the number of neighbourhoods found for all three classes as a function of population. For each class, The curve is well-fitted by a powerlaw function of the form

$$\begin{array}{c} N_{n}=b\;H^{\tau } \end{array}$$ (12)

where the exponent *τ* is less than one and depends on the class, indicating that there are proportionally less neighbourhoods in larger cities (the number areal units scales proportionally with the population size). In other words, different classes become more spatially coherent as the population increases (see S1 Text for more details). The values of the exponents are

$$\begin{array}{ccc} \tau_{H} & = & 0.65\\ \tau_{L} & = & 0.73\\ \tau_{M} & = & 0.84 \end{array}$$

These values show that the tendency to cluster as the city size increases is larger for higher-income households than for lower- and middle-income households. In other words, segregation increases with city size.

## 4 Discussion

In this paper, we propose a general conceptual framework in which residential segregation can be quantified and understood. Instead of enumerating its different aspects, we took a radically different—yet simpler—approach. We define segregation by what it is not: a random distribution of the different households throughout the urban space. This naturally leads to define the measure of representation, which is used in turn to improve upon previous ways [48] to define neighbourhoods. We further define the exposure (still based on the representation), which measures the extent to which different categories attract, repel or are indifferent to one another.

We show that we can define classes in a non-parametric way and 3 main income classes emerge for the 2014 American Community Survey data. The middle-income class corresponds to a smaller income range than what is usually admitted, a curiosity that certainly deserves further investigations. These complex systems can thus be described by considering a small number of categories only. This is an important piece of information which will simplify the description and modelling of stratification mechanisms.

In terms of spatial arrangement, although the fraction of the population that is contained in neighbourhoods does not change with city size, the neighbourhoods are geographically more coherent as cities get larger, which corresponds in effect to an increased level of segregation as the city size increases.

Our results also point to the intriguing fact that higher-income households are on average overrepresented in very dense areas. Such high density areas are relatively rare in the US, which might explain in part why we observe poor centers and rich suburbs and rich centers essentially in Europe where the density is very large. This result echoes Jacobs’ analysis [3] that neighbourhoods with the highest dwelling densities are usually the ones exhibiting the largest vitality, and are therefore the most attractive. Obviously, a high density is not the only determinant and in some cases high-density neighbourhoods can also be lower-income neighbourhoods. Further investigations along these lines may provide quantitative insights into the mechanisms leading to urban decline or urban regeneration.

In this study, we also have tried to highlight the *spatial* pattern of segregation. We believe that the identification of neighbourhoods that our method permits will allow a finer-scale investigation of these spatial patterns. However, the fundamental issue that runs beneath—the need for new tools in the analysis of spatial patterns—is still open. It goes beyond the problem of segregation and has a huge number of potential applications.

## 5 Materials and methods

### 5.1 Data

In this study, we use the American Census Bureau’s 2014 American Community Survey data on the income of households at the census block-group level, grouped per Core-based Statistical Areas. The households are divided in 16 income categories, ranging from below $10,000 annual income to above $200,000. All data of the 2014 American Community Survey are available from the Census Bureau. 2140 delineations of the Core-based Statistical Areas are available from the Office of Budget Management. The reader interested in obtaining a cleaned version of these data ready for analysis and/or reproduce the results of this analysis can consult the online repository (The code necessary to download, assemble the data and reproduce the analysis performed in this article is freely available online at http://github.com/rlouf/patterns-of-segregation).

### 5.2 Software

The methods described in this manuscript are very general, and not limited to the study of income segregation. In order to facilitate their application to other datasets, all the measures have been packaged in a python library, Marble, open-source and freely available online [25].

## Supporting Information

S1 Text Calculations and procedures. (PDF)
