# Supplementary material for: Patterns of Residential Segregation
Source: PLoS One. 2016 Jun 17;11(6):e0157476. doi: 10.1371/journal.pone.0157476 (PMC4912131; doi:10.1371/journal.pone.0157476)
Supplement: S1 Text — (PDF) [file pone.0157476.s001.pdf]

Supporting Information
Patterns of residential segregration

Rémi Louf and Marc Barthelemy
Institut de Physique Théorique, CEA, CNRS-URA 2306, F-91191, Gif-sur-Yvette, France

CONTENTS

Exposure 2
Definition 2
Expected value and variance in the random configuration 3
Minimum and maximum values 3
Isolation I_α 3
Exposure $E_{\alpha\beta}$ 4

Agregating categories into classes 5
Method 5
Computing the class structure at the country scale 5
Results 6

Larger cities are richer and more unequal 6

Proportion of households in neighbourhoods 8

Number of over-represented units and city size 8

References 9

The following supporting information provide more details on the calculations made to obtain the maximum and minimum value of exposure and isolation, and their variance. We also detail the process through which we agregate the original categories into classes, and the results we obtain for the 2014 American Community Survey. Finally, we discuss in more details some of the results presented in the manuscript; in particular, our claim that larger cities are richer.

EXPOSURE

Definition

Given two different categories α and β, we define their intra-unit exposure as the average representation r_α (resp. r_β) of the α (resp. β) that is seen on average by the members of β (resp. α)

$$E_{\alpha\beta} = \frac{1}{N_\alpha} \sum_t n_\alpha(t)\, r_\beta(t) \tag{1}$$

It can also be re-written

$$E_{\alpha\beta} = \frac{1}{N} \sum_{t=0}^{T} n(t)\, E_{\alpha\beta}(t) \tag{2}$$

where

$$E_{\alpha\beta}(t) = r_\alpha(t)\, r_\beta(t) \tag{3}$$

This expression symmetric by permutation of the categories α and β, which is what one would expect from an index measuring the interaction between two categories.

Expected value and variance in the random configuration

In order to know whether the attraction or repulsion mesured between two classes is significant, we need to be able to compute $\text{Var}\left[E_{\alpha\beta}\right]$. Assuming that $r_\alpha(t)$ and $r_\beta(t)$ are independent (which is rigorously not true for tracts with a fixed capacity $n(t)$), it follows

$$\begin{aligned}\text{E}\left[E_{\alpha\beta}(t)\right] &= \text{E}[r_\alpha(t)]\text{E}[r_\beta(t)] = 1\\ \text{Var}[E_{\alpha\beta}(t)] &= \frac{1}{N_\alpha N_\beta}\left(\frac{N}{n(t)}-1\right)^2 + \frac{1}{N_\alpha}\left(\frac{N}{n(t)}-1\right) + \frac{1}{N_\beta}\left(\frac{N}{n(t)}-1\right)\end{aligned}$$

Thus

$$\begin{aligned}\text{E}[E_{\alpha\beta}] &= 1\\ \text{Var}[E_{\alpha\beta}] &= \frac{1}{N^2}\sum_t n(t)^2\,\text{Var}[E_{\alpha\beta}(t)] + \frac{2}{N^2}\sum_{s<t} n(s)\,n(t)\,\text{Cov}[E_{\alpha\beta}(s), E_{\alpha\beta}(t)]\end{aligned}$$

The covariance is non-zero because the $n_\alpha(t)$ of two different tracts t and s are not independent, and we have

$$\text{Cov}[E_{\alpha\beta}(s), E_{\alpha\beta}(t)] = \left(1-\frac{1}{N_\alpha}\right)\left(1-\frac{1}{N_\beta}\right) - 1 \tag{4}$$

Minimum and maximum values

In order to be able to make sense of the values of exposure ($E_{\alpha\beta}$) and isolation (I_α), and compare different cities, we need to know their respective maximum and minimum values. We will consider the following cases:

Maximum isolation: Situation where each areal unit contains households from one and only one category. This situation corresponds to the minimum of $E_{\alpha\beta}$ and the maximum of I_α.

The unsegregated city: When the distribution of households in the different areal units cannot be distinguished from a random distribution. This is what we call the ‘unsegregated city’ and gives a point of reference. It corresponds to the minimum of I_α.

Isolation I_α

In the unsegregated city case, there is no way to tell the difference between the distribution of the different categories in the different tracts and a random distribution. In this situation, isolation indices I_α reach their minimum value

$$\boxed{I_\alpha^{min} = 1}$$

when $r_\alpha(t) = 1$, $\forall\, t$.

In the maximum isolation case, all categories are alone in their own tract. In other words, $\forall\, t$ and $\forall\, \beta \neq \alpha$ we have $n_\beta(t) = 0$ iff $n_\alpha(t) \neq 0$. We thus obtain for the isolation

$$\begin{aligned}I_\alpha &= \frac{1}{N_\alpha}\sum_{t=1}^{T} n_\alpha(t)\,r_\alpha(t)\\ &= \frac{1}{N_\alpha}\sum_{t\in\mathcal{R}_\alpha}\frac{n_\alpha(t)^2}{n(t)}\frac{1}{N_\alpha/N}\\ &= \frac{N}{N_\alpha^2}\sum_{t\in\mathcal{R}_\alpha}\frac{n_\alpha(t)^2}{n(t)}\end{aligned}$$

where $\mathcal{R}_\alpha$ is the set of areal units where the category α is present. In these unit, $n(t) = n_\alpha(t)$. Therefore

$$\boxed{I_\alpha^{max} = \frac{N}{N_\alpha}}$$

Exposure $E_{\alpha\beta}$

In the maximum isolation case, all categories are alone in their own tract. In other words, $\forall\, t$ and $\forall\, \beta \neq \alpha$ we have $n_\beta(t) = 0$ iff $n_\alpha(t) \neq 0$. In this situation, we trivially have

$$\boxed{E_{\alpha\beta}^{min} = 0}$$

The maximum of the exposure is however more difficult to obtain in general. We fix α and β and we denote by a category γ all the rest. By definition we have $\sum_t n_\alpha(t) = N_\alpha$, $\sum_t n_\beta(t) = N_\beta$, and $\sum_t n_\gamma(t) = N - N_\alpha - N_\beta$.

We will look for the 'global' maximum by keeping the only constraint that in each unit we have $n(t) = n_\alpha(t) + n_\beta(t) + n_\gamma(t)$. We obtain for the exposure

$$E_{\alpha\beta} = \frac{N}{N_\alpha N_\beta} \sum_t \frac{n_\alpha(t)(n(t) - n_\alpha(t) - n_\gamma(t))}{n(t)} \tag{5}$$

The maximization of the exposure with respect to $n_\alpha(t)$ thus gives

$$\frac{\partial E_{\alpha\beta}}{\partial n_\alpha(t)} = 0 = \frac{N}{N_\alpha N_\beta}\left[n(t) - n_\gamma(t) - 2n_\alpha(t)\right] \tag{6}$$

which leads to

$$n_\alpha^*(t) = \frac{n(t) - n_\gamma(t)}{2} \tag{7}$$

The exposure for these values reads

$$E_{\alpha\beta}(\{n_\alpha^*\}, \{n_\gamma\}) = \frac{N}{N_\alpha N_\beta} \sum_t \frac{(n(t) - n_\gamma(t))^2}{4n(t)} \tag{8}$$

The quantity $n_\gamma(t)$ is in the compact set $[0, n(t)]$ and the maximization is not necessarily given by taking the derivative equal to zero. Indeed, in this case the maximum of $E_{\alpha\beta}(\{n_\gamma\})$ is obtained for $n_\gamma(t) = 0$ for all t (while the derivative equal to zero would lead to the minimum obtained for $n_\gamma = n(t)$ for all t) and reads

$$\boxed{E_{\alpha\beta}^{max} = \frac{N^2}{4N_\alpha N_\beta}} \tag{9}$$

This maximum is the global one, obtained when there are no constraints. One can easily add the constraint $\sum_t n_\alpha(t) = N_\alpha$ by using a Lagrange multiplier λ and we have then to maximize the function

$$E_{\alpha\beta} = \frac{N}{N_\alpha N_\beta} \sum_t \frac{n_\alpha(t)(n(t) - n_\alpha(t) - n_\gamma(t))}{n(t)} - \lambda(\sum_t n_\alpha(t) - N_\alpha) \tag{10}$$

The derivative with respect to $n_\alpha(t)$ leads to

$$n_\alpha^*(t) = \frac{1}{2}\left(n(1 - \frac{N_\beta - N_\alpha}{N}) - n_\gamma\right) \tag{11}$$

where we expressed the constraint $\sum_t n_\alpha(t) = N_\alpha$ in order to eliminate the Lagrange multiplier λ. We can then express the maximum $E_{\alpha\beta}(\{n_\gamma\})$ obtained for these values of n_α and as above the maximum is obtained for $n_\gamma(t) = 0$ for all t and reads

$$E_{\alpha\beta}^{max,c} = \frac{N^2}{4N_\alpha N_\beta}\left[1 - \left(\frac{N_\beta - N_\alpha}{N}\right)^2\right] \tag{12}$$

which is obviously smaller than the global maximum $E_{\alpha\beta}^{max}$.

These maxima were obtained when there are no constraints on the total number $\sum_t n_\gamma(t)$. When there is such a constraint, the construction of the maximum of Eq. (8) is not trivial. Very likely, when $\sum_t n_\gamma(t) = N_\gamma$ is fixed, we have to fill the smallest tracts with this class γ and we are then left with the classes α and β only. It seems difficult to obtain an analytical derivation of this maximum and we will keep as a reference in our calculations the global maximum $E_{\alpha\beta}^{max}$.

AGREGATING CATEGORIES INTO CLASSES

The study of segregation must be rooted in a particular definition of class. However, the income is a continuous variable, and there is no clear definition of incomes classes in the litterature: a class means different things to different people. We thus start by finding out the class structure as it manifests itself in the spatial arrangement of people.

Method

We take as a starting point the finest income subdivision given by the Census Bureau (16 subdivisions) and compute the 16×16 matrix of $E_{\alpha\beta}$ values at the scale of each cities. We then perform hierarchical clustering on this matrix, successively aggregating the subdivisions with the highest $E_{\alpha\beta}$ values. The process, implemented in the library Marble [S2], goes as follows:

1. Check whether there exists a pair α, β such that $E_{\alpha\beta} > 1 + 10\,\sigma$ (i.e. two categories that attract one another with at least 99% confidence according to the Chebyshev inequality). If not, stop the agregation and return the classes;

2. If there are some couples satisfying (1), normalize all $E_{\alpha\beta}$ values by their respective maximum values. Find then the pair γ, β whose normalized exposure is the maximum;

3. Aggregate the two categories β and γ;

4. Restart the process until it stops.

In order to aggregate the categories at step 3, we need to compute the distance betweence $\delta = \beta \cup \gamma$ and any category α once β and γ have been aggregated. Using the definition of $E_{\alpha\beta}$, it is easy to show that

$$E_{\alpha\delta} = \frac{1}{N_\beta + N_\gamma}\left(N_\beta\, E_{\alpha\beta} + N_\gamma\, E_{\alpha\gamma}\right) \tag{13}$$

The variance is also easily calculated as:

$$\mathrm{Var}\left[E_{\alpha\delta}\right] = \frac{1}{\left(N_\beta + N_\gamma\right)^2}\left(N_\beta^2\,\mathrm{Var}\left[E_{\alpha\beta}\right] + N_\gamma^2\,\mathrm{Var}\left[E_{\alpha\gamma}\right]\right) \tag{14}$$

Computing the class structure at the country scale

We computed the class structure at the scale of the whole US. We assume that the country is a juxtaposition of the different cities, with independent values of $E_{\alpha\beta}^c$. We then compute the average over the whole country and obtain

$$E_{\alpha\beta}^{US} = \frac{1}{N_{US}}\sum_c N_c\, E_{\alpha\beta}^c \tag{15}$$

where N_c is the population of the city c, and N_{US} the urban population of the US. The sum runs over all MSAs in the US. The variance is then given by

$$\mathrm{Var}\left[E_{\alpha\beta}^{US}\right] = \frac{1}{N_{US}^2}\sum_c (N_c)^2\,\mathrm{Var}\left[E_{\alpha\beta}^c\right] \tag{16}$$

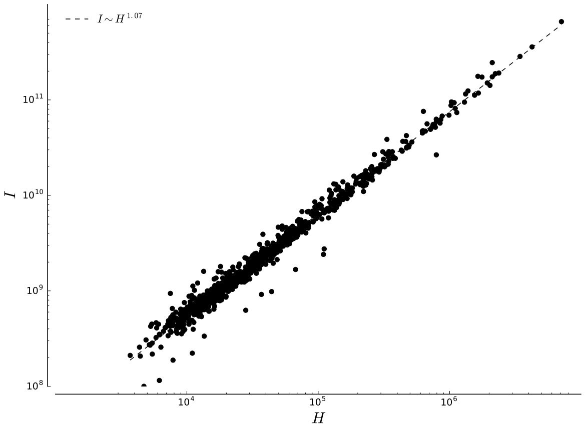

FIG. 1. Total income of households versus the total number of households. The dashed line represents a power-law fit with exponent 1.08 ($R^2 = 1$).

Results

Starting from categories 0 (the poorest) to 15 (the wealthiest), our methods finds the following classes for the US

(L - 59%) 0|1|2|3|4|5|6|7|8
(M - 11%) 9|10
(H - 29%) 11|12|13|14|15

with in parenthesis the percentage of the total US population that is included in the corresponding classes.

LARGER CITIES ARE RICHER AND MORE UNEQUAL

Although intuitively appealing, the idea that larger Metropolitan areas are richer is not as straightforward as it seems. The first question one can ask is if people are richer on average in large cities ? As shown in Fig. 1, the total income in a city scales (slightly) superlineary with population size

$$I \sim P^{1.07} \tag{17}$$

which suggests that the income *per household* is on average higher in larger cities than in smaller ones. In other words, there are proportionally more households belonging the to the wealthiest categories in large cities. In other words, the income inequality is higher in large cities than in small ones.

In order to measure levels of income inequality, we compute the Gini coefficient of the income distribution for every Core-based Statistical Area using the formula proposed in [S1]

$$G = \frac{1}{2\,N(N-1)\,\overline{I}} \sum_{i,j=1}^{H} |I_i - I_j| \tag{18}$$

The results, shown in Fig. 2 do not show any dependence of the Gini coefficient on the metropolitan population. This example shows that the Gini coefficient is not always a good measure of inequality and can be too aggregated to detect finer details. In order to confirm the consequence of the superlinear scaling of income in terms of larger cities having proportionally more higher-income households, we plot the number of households belonging to the 3 different classes as a function of the total number of households on Fig. 3. We find that for three classes, the data are well

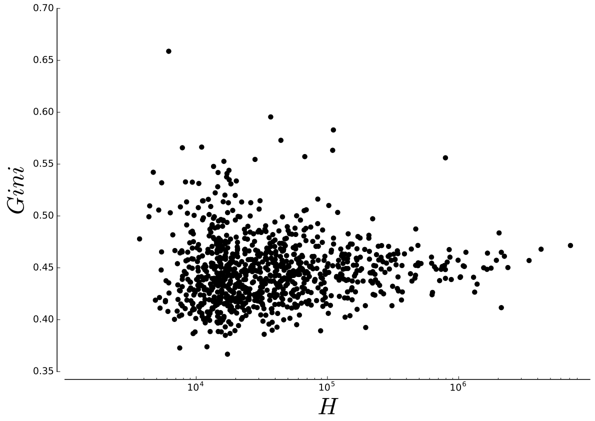

FIG. 2. Gini coefficient of the income distribution of the 280 MSA in 2014 versus the number of households in the city. No clear trend can be observed here.

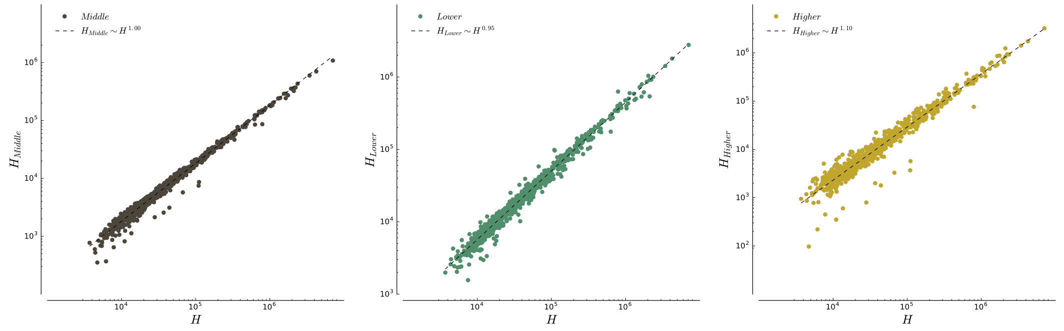

FIG. 3. Scaling of the number of households in each class with the total number of households for the 2014 Core-based Statistical Areas. The fits are extremely good with a $R^2 > 0.98$ in all three cases.

approximated by a power-law relationship

$$H_L \sim H^{0.95}$$
$$H_M \sim H^{1.00}$$
$$H_H \sim H^{1.10}$$

The problem with writing scaling relationships in this case is that it the constraint $H_L + H_M + H_H = H$ is hidden (ie. the numbers of households belonging to each category must sum to the total number of households). We therefore write

$$H_i = \eta_i(H)\, H \tag{19}$$

where η_i is the fraction of households in the city that belong to the class i. The constraint that the numbers of households in each class should sum to H is equivalent to

$$\eta_L + \eta_M + \eta_H = 1 \tag{20}$$

We plot these ratios on Fig. 4 and we indeed see that the number of households belonging to the higher-income class is proportionally larger in larger cities (for $H > 2 - 300,000$), while the number of households belonging to the lower-income class is proportionally smaller. The proportion of Middle-income class households stays essentially the same across all metropolitan areas.

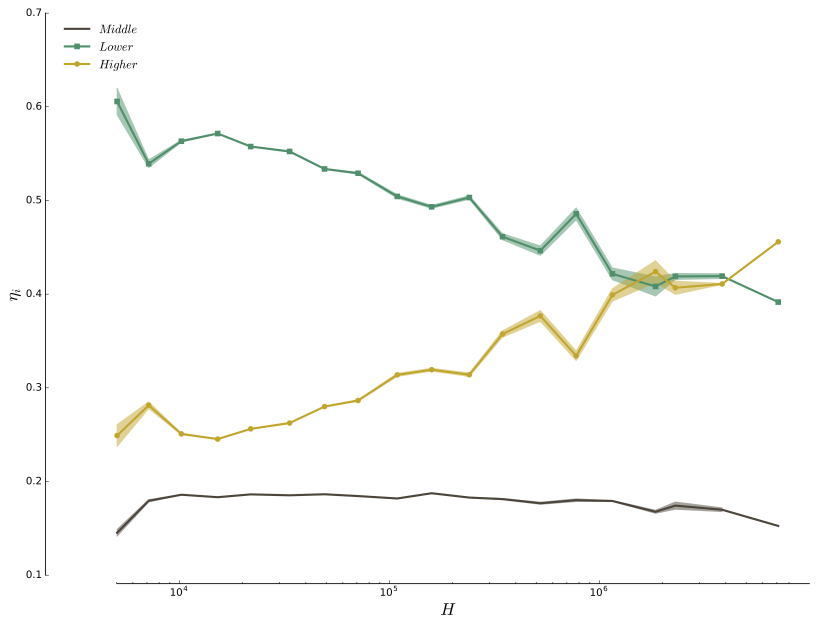

FIG. 4. Fraction η_i of the number of households belonging to the class i in cities versus the total number H of households. The curve shows the average values obtained when binning per household (the shaded area represent the standard deviation around this average). The proportion of middle-income classes stays sensibly constant with respect to population. In contrast, the order of the lower and higher income fractions is inverted when the population crosses $2 - 300,000$ invidivuals.

In this work, we take a different approach and ask if the different classes are more or less represented in a given MSA, compared to the average US result. In this context, a city is richer if the higher-income class is over-represented in this city, while the lower-income class is under-represented. The measure stems from the realisation that 'rich' and 'poor' are not absolute concepts, but must be related to the environment. In this case, it makes sense to compare the representation of the different income classes between metropolitan areas.

PROPORTION OF HOUSEHOLDS IN NEIGHBOURHOODS

Neighbourhoods identify the areas in the city where the categories are overrepresented, but this does not necessarily mean that most households belonging to a category live in either of the corresponding neighourhood. We plot the distribution of the proportion of households belonging to the lower-, middle- and higher- income classes that also live in a corresponding neighbourhood on Fig. 5.

One can see that higher-income households tend to be more concentrated in the regions where they are represented, with an average of 52%. Followed by the lower-income households, with and average of 40%. The middle-income households are equally evenly spread across the city, with an average of 40%.

NUMBER OF OVER-REPRESENTED UNITS AND CITY SIZE

In the main text, we find that the number of neighbourhoods for the 3 classes grows sublinearly with the size of a city, with a behaviour that is well approximated by a power-law

$$N_n \sim H^{\nu} \tag{21}$$

with $\nu = 0.86$ ($r^2 = 0.97$) for all classes together. We claim this shows the tendency of classes to cluster more in larger cities than in smaller ones. This is only true, however, if the number of areal units in which each class is overrepresented does not itself vary sublinearly with population size. We plot on Fig. 6 these numbers for each class

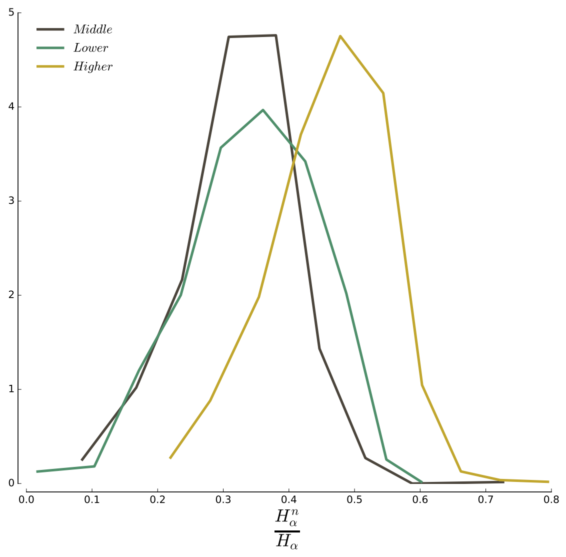

FIG. 5. Distribution of the fraction of households belonging to a given class and that live in a neighborhood where it is over-represented (Middle, Lower, or Higher).

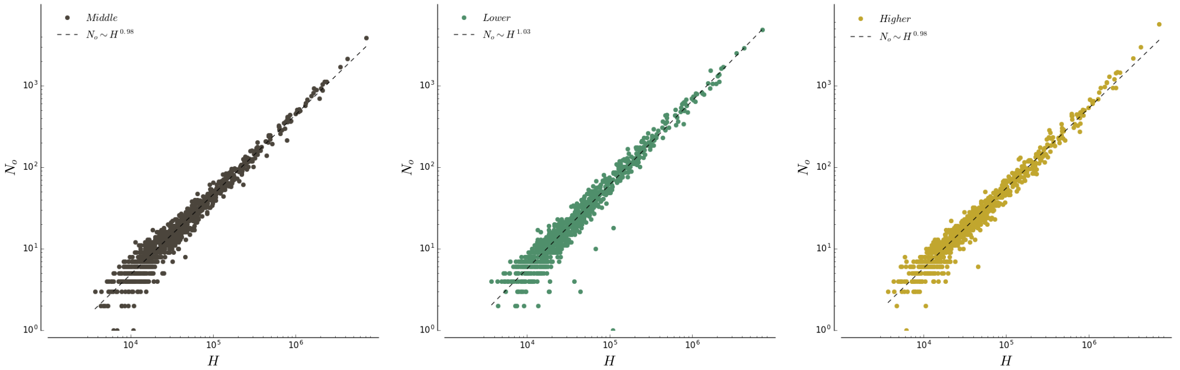

FIG. 6. Number of areal units where each class is overrepresented as a function of the total number of households in the city. The behaviour is consistent with a linear behaviour in the three cases.

and each city as a function of the size of the city. We find that the behaviour of the number of overrepresented units is consistent with a linear behaviour for all three classes

$$N_o \sim H \tag{22}$$

And our claim of increased clustering is thus justified.

[S1] Dixon, PM et al. (1987) Bootstrapping the Gini coefficient of inequality. *Ecology* 1548-1551.
[S2] Louf, R (2015) Marble, a python library to study patterns of segregation. Code and documentation freely available online at http://github.com/scities/marble
